# A Novel Depression Risk Prediction Model Using NHANES Data With Mendelian Randomization Validation

**DOI:** 10.1002/brb3.70674

**Published:** 2025-07-25

**Authors:** Lin Lin, Liqun Zhang, Jingdong Zhang, Dapeng Ding

**Affiliations:** ^1^ Department of Clinical Laboratory Medicine First Affiliated Hospital of Dalian Medical University, Zhongshan Road, Xigang District Dalian Liaoning Province China; ^2^ School of Biomedical Engineering, Faculty of Medicine Dalian University of Technology, No. 2 Linggong Road, Ganjingzi District Dalian Liaoning Province China; ^3^ Medical Oncology Department of Gastrointestinal Cancer, Liaoning Cancer Hospital, & Institute Cancer Hospital of China Medical University, No. 44 Xiaoheyan Road, Dadong District Shenyang Liaoning Province China

**Keywords:** biochemical markers, depression, Mendelian randomization (MR), national health and nutrition examination survey (NHANES), predictive modeling

## Abstract

**Background:**

Despite depression's significant public health impact, efficient and accessible screening tools utilizing routine clinical indicators remain limited. This study aimed to develop and validate a practical depression risk prediction model based on commonly available biochemical markers, facilitating widespread early screening and timely intervention in general clinical settings.

**Methods:**

We formulated a model for depression, scrutinizing an assortment of biochemical indicators and their bidirectional interrelationships with depression, employing data derived from the National Health and Nutrition Examination Survey (NHANES) and leveraging the Mendelian randomization (MR) approach, a method that utilizes genetic variants as instrumental proxies to ascertain causal nexus between risk determinants and diseases.

**Results:**

Using NHANES data (training cohort: n = 27,327; validation cohort: n = 4383), we developed two prediction models through LASSO and multivariate logistic regression. Both models demonstrated comparable performance in terms of discrimination (ROC curves), calibration (slope and Hosmer‐Lemeshow test), Brier score, decision curve analysis, net reclassification improvement, and integrated discrimination improvement. Given the similar performance metrics and more parsimonious nature, Model 2, with 14 variables, was selected as the final model. MR analysis revealed bidirectional relationships between biomarkers and depression. Higher body mass index level was associated with increased depression risk (odds ratio [OR]: 1.061, *p* = 0.008). Depression itself showed significant associations with increased ALP (OR: 1.048, *p* = 0.010), decreased BUN (OR: 0.966, *p* = 0.032), and TB (OR: 0.963, *p* = 0.044) levels.

**Conclusions:**

Model 2, selected for its predictive accuracy and streamlined complexity, presents a pragmatic instrument for large‐scale population screenings, facilitating timely intervention and therapeutic strategies.

## Graphical abstract

This study develops and validates a novel depression risk prediction model by integrating machine learning analysis of the National Health and Nutrition Examination Survey (NHANES) dataset with a two‐sample Mendelian Randomization (MR) approach. The work provides key evidence in three primary areas: the development of a parsimonious 14‐variable predictive model for large‐scale depression risk screening using routine biochemical and demographic markers; the identification of a causal relationship where higher body mass index (BMI) increases the risk of depression; and the elucidation of causal effects of depression on several biomarkers, including increased alkaline phosphatase (ALP) and decreased blood urea nitrogen (BUN) and total bilirubin (TB) levels.

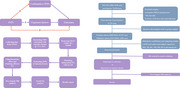

## 1 Introduction

Depression, a ubiquitous and incapacitating psychiatric condition, afflicts over 35 million individuals globally. The recent pandemic has dramatically exacerbated this depressive toll; according to the 2020 Global Burden of Disease data drawn from 204 nations, throughout 2020, COVID‐19 and associated lockdowns precipitated a 27.6% escalation in depression cases (Santomauro et al. 2021).

Depression exists within a complex network of interconnected mental health conditions. Sleep disorders, anxiety, and stress frequently co‐occur with depression, forming an intricate web of psychological and physiological manifestations (Bin Heyat et al. 2022; Heyat et al. 2024). Recent studies have highlighted the role of oxidative stress and inflammation in both depression and sleep disorders, suggesting shared biological pathways (Fazmiya et al. 2022). The relationship between anxiety and depression has been extensively documented, with both conditions showing similar patterns of hormonal and genetic influences.

Recent years have witnessed significant advances in depression detection methodologies, particularly in the realm of artificial intelligence. Machine learning models have been successfully applied to various physiological signals, including electrocardiogram data, for detecting mental stress and related conditions (Pal et al. 2022; Akhtar et al. 2024; Heyat et al. 2019; Bin Heyat et al. 2020; Heyat et al. 2021; Bin Heyat et al. 2021). Innovative approaches using wearable devices and the internet of medical things have shown promise in mental stress detection (Bin Heyat et al. 2022). These technological advances, while promising, often require specialized equipment and controlled environments for monitoring, limiting their applicability for large‐scale population screening in routine clinical settings. Moreover, the cost and accessibility of such specialized devices may restrict their widespread implementation in general healthcare facilities.

Similarly, conventional questionnaire‐based detection methodologies, which rely heavily on self‐reported symptoms, present their own challenges. These subjective measures are susceptible to individual interpretation variances, potential nondisclosure, and both intentional and unintentional misrepresentation. Furthermore, a substantial portion of the population may either be reluctant to participate in depression screening questionnaires or dismiss their symptoms due to stigma and other sociocultural factors (Saitz et al. 2014).

These limitations highlight the need for an alternative approach that is both objective and readily implementable in routine healthcare settings. Recent studies have revealed promising associations between depression and various blood biomarkers, including albumin, high‐density lipoprotein (HDL), alkaline phosphatase (ALP), and white blood cell (WBC) count (Cao et al. 2022; Zhang et al. 2020; Liang et al. 2023; Wu et al. 2021). These objective physiological indicators not only show potential causal relationships with depression but also offer several practical advantages: they are routinely collected during regular health check‐ups, require no specialized equipment beyond standard clinical laboratory facilities, and can be easily integrated into existing healthcare workflows. This suggests an opportunity to develop a more accessible and objective approach to depression screening by leveraging these commonly available biochemical markers.

The comprehensive National Health and Nutrition Examination Survey (NHANES) database could serve as a robust data repository, enabling us to formulate a model that utilizes indicators to forecast depression risk. Moreover, to enrich our investigation, we conducted a Mendelian randomization (MR) analysis on the variables derived from our model. MR is an ascendant epidemiology technique that utilizes genetic variables as surrogate markers for exposure (e.g., blood biomarkers) to estimate causal impacts on a specific outcome (e.g., depression). This method is less susceptible to bias from confounding factors and reverse causality compared to observational studies (van Kippersluis and Rietveld 2018). This pioneering approach enables us to infer potential causal associations between these markers and the risk of depression, which imparts a more profound understanding of the underlying biological mechanisms of depression.

## 2 Methods

### 2.1 Participant Selection From NHANES

NHANES, an ongoing biennial nationally representative study in the United States, is designed to scrutinize the health and nutritional status of the country's populace. Annually, the survey engages approximately 5000 individuals, employing a combination of interviews, physical examinations, and laboratory assays. The study protocol for NHANES was sanctioned by the National Centers for Health Statistics, and informed consent was procured from each participant. In the present study, we drew upon the 2005–2018 cycles of NHANES, as these seven iterations provided a comprehensive set of hematological indices as well as the Patient Health Questionnaire‐9 (PHQ‐9). Inclusion in the study necessitated completion of the PHQ‐9 questionnaire. Due to the open availability of NHANES, no ethical review is required.

### 2.2 Depression Assessment and Sample Collection Protocol

The NHANES depression screening protocol follows a standardized two‐phase approach. Initially, participants complete the PHQ‐9 either in their homes or in mobile examination centers under the supervision of trained interviewers. The PHQ‐9 is administered as part of the mental health depression screener interview, which is conducted in a private setting to ensure confidentiality and accurate reporting.

Depression status within our study was assessed utilizing the validated PHQ‐9, exhibiting a Cronbach's alpha of 0.89, indicating excellent internal consistency and reliability of the depression assessment in our study population. The individual symptom items within the PHQ‐9 are gauged on a four‐tiered Likert scale, ranging from 0 (‘not at all’) to 3 (‘nearly every day’), yielding an aggregate score bracket of 0 to 27. Stratification of participants was predicated on their total score, categorizing them into groups with either negligible depression risk (0–4), or potential susceptibility to depression (5–27) (Kroenke et al. 2001).

Other parameters were collected during the same examination period as the PHQ‐9 administration, ensuring temporal concordance between biomarker measurements and depression assessment. This synchronous collection of physiological and psychological data allows for a more accurate representation of the relationship between biomarkers and current depression status, rather than historical depression episodes.

The NHANES protocol specifically emphasizes the assessment of current depressive symptoms rather than lifetime history, as this approach provides a more precise snapshot of the physiological‐psychological intersection at a specific point in time. This methodology aligns with our research objective of identifying real‐time biological markers that could serve as objective screening tools for current depression risk.

### 2.3 Data Extraction From NHANES

Our study takes a comprehensive approach by incorporating a broad spectrum of demographic characteristics, anthropometric measurements, and hematological parameters. This extensive inclusion strategy is rooted in the complex, multifaceted nature of depression, which involves various physiological and biochemical pathways. By casting a wider net in our initial variable selection, we aim to capture potential interactions and relationships that might be overlooked in more narrowly focused analyses. This approach allows us to account for the intricate interplay between different physiological systems and their collective influence on depression risk, potentially revealing novel biomarker combinations that could enhance screening accuracy. Data was collated only for participants aged 18 years and older and included parameters such as height, weight, and body mass index (BMI), along with a comprehensive blood profile, including complete blood count and standard biochemistry profile. Variables with missing data less than 5% were handled using multiple imputation, while those exceeding this threshold were excluded from the study. Consequently, the study encapsulated the following metrics: age (years), gender (male or female), race (Mexican American, Other Hispanic, Non‐Hispanic White, Non‐Hispanic Black, and Other Race), height (cm), weight (kg), BMI (kg/m^2^), glucose (mmol/L), cholesterol (mmol/L), triglycerides (mmol/L), HDL (mmol/L), ALP (U/L), alanine transaminase(ALT, U/L), aspartate aminotransferase (AST, U/L), gamma‐glutamyl transferase (GGT, U/L), lactate dehydrogenase (LDH, U/L), total bilirubin (TB, µmol/L), albumin (g/L), globulin (g/L), total protein (g/L), creatinine (µmol/L), BUN (mmol/L), uric acid (µmol/L), iron (µmol/L), sodium (mmol/L), potassium (mmol/L), chloride (mmol/L), bicarbonate (mmol/L), calcium (mmol/L), phosphorus (mmol/L), osmolality (mmol/kg), WBC count (1000 cells/µL), neutrophils number (1000 cells/µL), neutrophils percent (%), lymphocyte number (1000 cells/µL), lymphocyte percent (%), monocyte number (1000 cells/µL), monocyte percent (%), platelet count (1000 cells/µL), mean platelet volume (fL), eosinophils number (1000 cells/µL), eosinophils percent (%), basophils number (1000 cells/µL), basophils percent (%), red blood cell (RBC) count (million cells/µL), hemoglobin (g/L), hematocrit (%), Mean cell hemoglobin concentration (MCHC, g/dL), mean cell hemoglobin (MCH, pg), mean cell volume (MCV, fL), red cell distribution width (RDW, %).

Participants presenting with severe aberrations in their indices, potentially indicative of serious pathologies, were excluded from this study. However, we deliberately retained samples from patients with moderate index abnormalities. This methodological decision was guided by our aim to develop models with broader real‐world applicability. While strict selective sampling approaches may yield more impressive statistical metrics, they potentially limit the generalizability of findings to real‐world clinical settings where patient populations are inherently heterogeneous and complex. Our inclusive sampling strategy thus prioritizes external validity and clinical utility over potentially inflated performance metrics derived from highly selected populations. We surmised that the spectrum of indices pertinent to this research is as delineated below: BMI (13–50 kg/m^2^), glucose (2.8–14 mmol/L), cholesterol (< 20 mmol/L), triglycerides (< 11.29 mmol/L), ALP (< 260 U/L), ALT (< 200 U/L), AST (< 175 U/L), GGT (< 225 U/L), LDH (< 1000 U/L), TB (< 85.5 µmol/L), albumin (≥ 30 g/L), globulin (10‐50 g/L), creatinine (< 177 µmol/L), BUN (< 20 mmol/L), iron (> 2 µmol/L), sodium (129‐150 mmol/L), potassium (2.9–6 mmol/L), chloride (90‐115 mmol/L), bicarbonate (18–33 mmol/L), calcium (1.75–3.5 mmol/L), phosphorus (0.32–1.78 mmol/L), WBC count (3‐20 × 1000 cells/µL), neutrophils number (1.1–12.6 × 1000 cells/µL), lymphocyte number (< 8 × 1000 cells/µL), platelet count (80–500 × 1000 cells/µL), eosinophils number (< 1.6 × 1000 cells/µL), basophils number (< 0.4 × 1000 cells/µL), RBC count (≥ 3 × million cells/µL), hemoglobin (≥ 90 g/L).

Finally, a total of 31,710 cases were included in this study. The detailed clinical characteristics of all participants are presented in Supplementary Table S1.

### 2.4 Development and Evaluation of Depression Risk Model Based on NHANES Dataset

We sectioned the NHANES data into chronological cohorts for training (2005–2016) and validation (2017–2018). As this study focused on developing a depression risk prediction model rather than conducting epidemiological research on depression, the subsequent statistical analyses and model development were performed using raw sample data from the NHANES dataset without applying NHANES‐specific sample weights.

When delineating disparities in variables between the training and validation cohorts, continuous variables were articulated through median and interquartile range (IQR), with categorical variables represented as frequencies and percentages. The Wilcoxon rank‐sum test and chi‐squared test were employed for continuous and categorical variables, respectively.

The present study deploys a dual methodology for model construction. Initially, due to the abundance of predictive variables, least absolute shrinkage and selection operator (LASSO) regression variable screening was deemed appropriate for the development group. LASSO regression filters lambda by implementing ten‐fold cross‐validation. Post LASSO regression, initial screening variables were examined for collinearity prior to modeling, with the exclusion criteria being variance inflation factor (VIF) values ≥ 5 (Tibshirani 1997). Subsequently, logistic regression analysis was performed to generate nationally representative estimates. Effect estimates were illustrated as odds ratio (OR) and 95% confidence interval (CI). Filtered variables in LASSO were substituted into the multivariate logistic regression analysis to produce model 1. We eliminated variables exhibiting a *p*‐value exceeding 0.05 from model 1, and performed a subsequent multivariate logistic regression analysis on the remaining variables to procure model 2.

Discriminative performance of the two models was evaluated using receiver operator characteristic (ROC) curves. The area under the curves (AUCs) was compared through the Delong method (*p*‐value > 0.05 on the Delong test infers no significant difference between AUC values) (DeLong et al. 1988). The Brier Score (BS, capturing calibration and discrimination aspects via mean squared deviation between predicted probabilities and actual outcomes) and Hosmer–Lemeshow (HL) goodness of fit test in the multiple logistic regression was employed for model calibration assessment (BS < 0.25 or HL p value > 0.05 implies a satisfactory overall model performance) (Hosmer et al. 2013), alongside the plotting of calibration curves. The net reclassification improvement (NRI) and integrated discrimination improvement (IDI) were calculated to estimate the discrimination accuracy of the prediction models (if NRI or IDI > 0 and *p*‐value < 0.05, the model improvement is significant) (Pencina et al. 2008; Kerr et al. 2011). The decision curve analysis (DCA) method was utilized to identify the maximal benefit of the prediction model (Van Calster et al. 2018).

Upon selecting the optimal model, the nomogram was based on proportionally translating each regression coefficient in the multivariable logistic regression to a 0 to 100‐point scale. The variable bearing the maximum β coefficient (absolute value) was assigned 100 points. These points were aggregated across independent variables to derive total points, subsequently converted to predicted probabilities (Lei et al. 2016).

### 2.5 The Data Resources of MR Analysis

To avoid potential bias from sample overlap in MR analyses, we obtained depression genome‐wide association studies (GWAS) data from the recently released FinnGen R12 dataset (November 4, 2024), which included 494,164 participants (59,333 cases and 434,831 controls) (Kurki et al. 2023). For testosterone, BMI, triglycerides, HDL, ALP, GGT, TB, albumin, BUN, WBC count, MCV, and RDW, we utilized GWAS data from the Pan‐ancestry Genetic Analysis of the UK Biobank, conducted by the Pan‐UK Biobank team. This analysis leveraged genetic and phenotypic data from approximately 500,000 UK Biobank participants (Bycroft et al. 2018). Detailed characteristics of each GWAS are presented in Supplementary Table S2.

### 2.6 The Design for the MR Analysis

**FIGURE 1:**
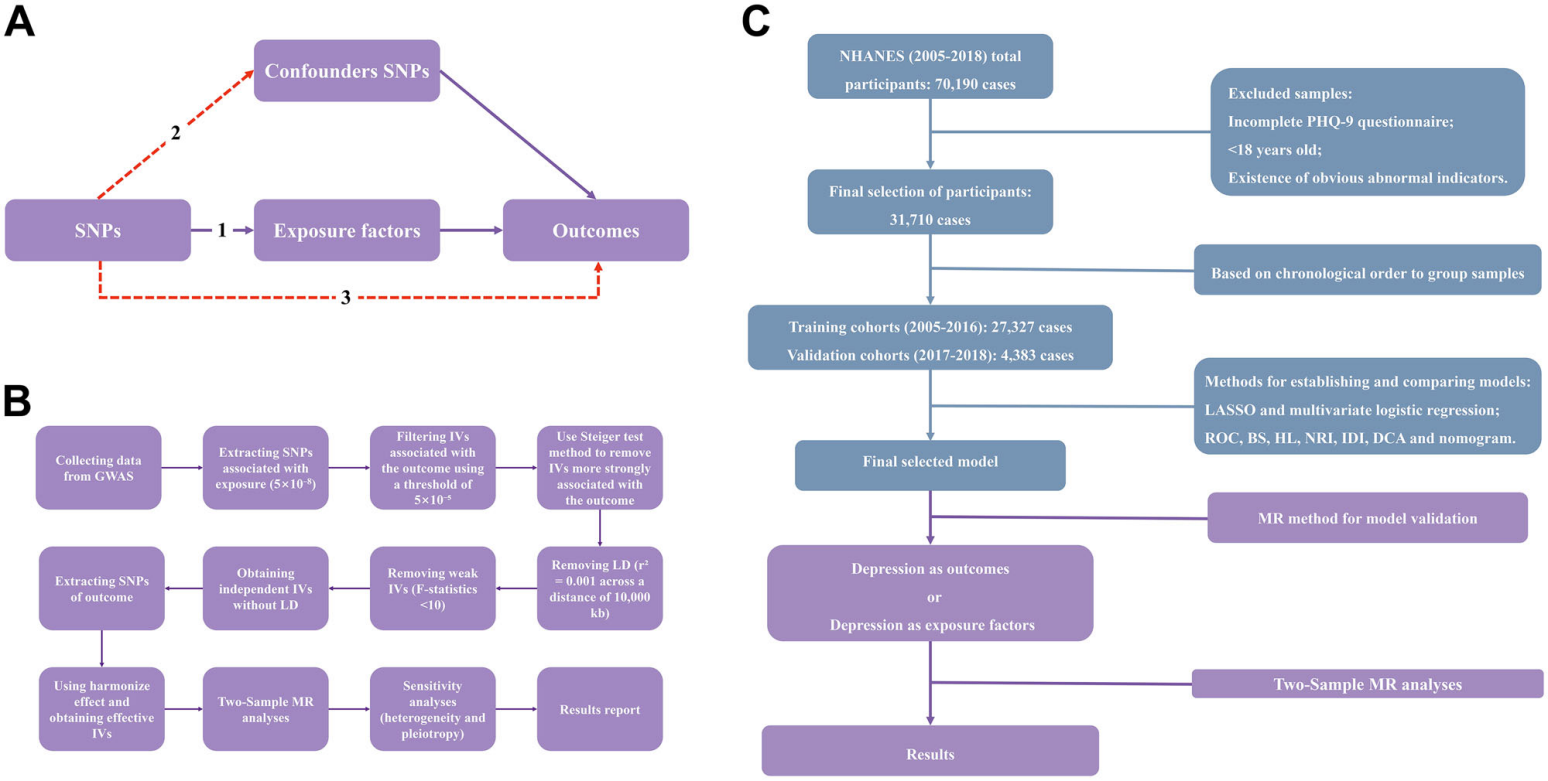
Study design and methodological framework. (A) Assumptions of Mendelian randomization, (B) two‐sample Mendelian randomization workflow, and (C) model development and validation pipeline.

This MR study was conducted and reported in accordance with the STROBE‐MR guidelines. We have ensured that our methodology, analysis, and reporting adhere to the best practices outlined in these guidelines to enhance the transparency, reproducibility, and reliability of our findings. As is shown in Figure 1A, our two‐sample MR analysis was grounded in three key assumptions: robust association between genetic instrumental variables (IVs) and exposure factors; independence from confounding variables; influence on outcomes exclusively through exposure factors (Smith and Hemani 2014; Holmes et al. 2017).

We implemented a rigorous, multi‐step process to ensure the validity of our MR analysis (Figure 1B). We sourced data from various GWAS. IVs were selected based on their significant association with exposure variables, using a genome‐wide significance threshold of 5 × 10⁻⁸. We filtered out IVs associated with the outcome using a threshold of 5 × 10⁻^5^. The Steiger test method was applied to evaluate the relative strength of each single nucleotide polymorphism (SNP)’s association with the exposure versus the outcome, ensuring selected IVs primarily influence the exposure rather than directly affecting the outcome (Hemani et al. 2017). This crucial step ensures that selected IVs primarily influence the exposure rather than directly affecting the outcome, thereby enhancing the validity of our MR analysis. To mitigate potential bias from linkage disequilibrium (LD), we removed SNPs in LD using a threshold of r^2^ = 0.001 across a distance of 10,000 kb (detailed in Supplementary Table S2) (Chang et al. 2015). We evaluated the strength of each IV using F‐statistics. F‐statistics were computed for each validated IV in both cohorts to negate the potential influence of weak IV bias using the formula:

(1) $$F=\frac{R^{2}\left(n-1-k\right)}{\left(1-R^{2}\right)k}\;$$

Here, n denotes sample size, k signifies the number of IVs, and R^2^ stands for the variance explained by the IVs (Pierce et al. 2011). Previous investigations have suggested an F statistic below 10 implies a weak IV, while an F‐value exceeding 11 may assure less than 10% relative bias at least 95% of the time, irrespective of the number of IVs deployed in the analysis (Pierce et al. 2011; Burgess and Thompson 2011). Weak IVs were subsequently excluded from further analysis. After obtaining a set of independent, non‐LD IVs, we extracted the corresponding SNPs for the outcome variable and applied the harmonize effect method to align effect sizes and alleles across exposure and outcome datasets. The detailed characteristics of selected IVs used in the MR analysis are presented in Supplementary Table S3.

We conducted our analyses using the “two sample MR,” “Mendelian randomization,” and “RadialMR” packages in R. The inverse‐variance weighted (IVW) method, implemented under a random‐effects model, served as our primary approach for estimating potential bidirectional causal relationships between exposure and outcome variables. This method assumes strict adherence to fundamental MR principles. We also employed MR‐Egger, weighted median, and weighted mode methods as complementary approaches, with results provided for reference. Although a significant IVW result was necessary, a positive finding was only considered valid when the direction of effect estimates was consistent across all analyses (IVW, MR‐Egger, weighted median, and weighted mode methods).

To ensure robust causal inference, we conducted sensitivity analyses, including assessments of heterogeneity and pleiotropy with a *p*‐value below 0.05 considered statistically significant (Bowden et al. 2015; Bowden et al. 2016). Heterogeneity was evaluated using both the IVW and MR‐Egger methods, interpreted via Cochran's Q‐test *p*‐value (Bowden et al. 2018). Horizontal pleiotropy was assessed using the Egger intercept method (Bowden and Holmes 2019). In this study, positive results were considered valid in the presence of heterogeneity but were excluded if horizontal pleiotropy was detected. Additionally, scatter plots, funnel plots, and leave‐one‐out analyses were generated to assess the robustness of positive findings.

The workflow of depression risk model development using the NHANES database and subsequent causal relationship assessment between included variables and depression risk using MR analysis is illustrated in Figure 1C. All aforementioned statistical analyses were executed utilizing R software (version 4.3.1; R Foundation for Statistical Computing, Vienna, Austria). All *p*‐values are two‐tailed, with a significance threshold of *p* < 0.05 for all analyses.

## 3 Results

### 3.1 Baseline Clinical Characteristics in NHANES

The baseline clinical characteristics of the study population are delineated in Table 1. Participants were bifurcated into two distinct cohorts: a training cohort comprising 27,327 individuals (84%), and a validation cohort comprising 4383 individuals (16%).

**TABLE 1:** The demographics and blood sample characteristics of the participants in NHANES.

Characteristic	Overall N = 31,710 (100%)^1^	Training cohort N = 27,327 (84%)^1^	Validation cohort N = 4,383 (16%)^1^	*p*‐value^2^
Risk of depression (n)	7579 (24%)	6496 (24%)	1083 (25%)	0.177
Age (years)	47.00 (31.00, 63.00)	46.00 (31.00, 63.00)	51.00 (33.00, 64.00)	< 0.001
Gender (n)				0.571
male	15,659 (49%)	13,512 (49%)	2147 (49%)	
female	16,051 (51%)	13,815 (51%)	2236 (51%)	
Race				< 0.001
Mexican American	5205 (16%)	4,580 (17%)	625 (14%)	
Other hispanic	3027 (9.5%)	2621 (9.6%)	406 (9.3%)	
Non‐hispanic white	13,648 (43%)	12,065 (44%)	1583 (36%)	
Non‐hispanic black	6410 (20%)	5466 (20%)	944 (22%)	
Other race	3420 (11%)	2595 (9.5%)	825 (19%)	
Height (cm)	167.10 (160.00, 174.60)	167.20 (160.20, 174.70)	166.30 (159.00, 173.90)	< 0.001
Weight (kg)	78.10 (66.20, 92.10)	78.00 (66.20, 91.90)	78.90 (66.60, 93.90)	0.005
BMI (kg/m^2^)	27.80 (24.12, 32.23)	27.70 (24.07, 32.09)	28.30 (24.60, 33.20)	< 0.001
Glucose (mmol/L)	5.11 (4.72, 5.72)	5.11 (4.72, 5.72)	5.16 (4.83, 5.66)	< 0.001
Cholesterol (mmol/L)	4.89 (4.22, 5.61)	4.89 (4.24, 5.64)	4.76 (4.11, 5.51)	< 0.001
Triglycerides (mmol/L)	1.33 (0.88, 2.04)	1.33 (0.88, 2.07)	1.30 (0.90, 1.90)	0.009
HDL (mmol/L)	1.32 (1.09, 1.60)	1.32 (1.09, 1.60)	1.32 (1.09, 1.58)	0.353
ALP (U/L)	67.00 (55.00, 81.00)	65.00 (54.00, 79.00)	75.00 (62.00, 90.00)	< 0.001
ALT (U/L)	20.00 (16.00, 28.00)	21.00 (16.00, 28.00)	18.00 (13.00, 25.00)	< 0.001
AST (U/L)	23.00 (19.00, 27.00)	23.00 (20.00, 28.00)	19.00 (16.00, 24.00)	< 0.001
GGT (U/L)	19.00 (14.00, 29.00)	19.00 (14.00, 29.00)	21.00 (14.00, 31.00)	< 0.001
LDH (U/L)	129.00 (113.00, 148.00)	125.00 (111.00, 143.00)	154.00 (137.00, 174.00)	< 0.001
TB (µmol/L)	10.26 (8.55, 13.68)	10.26 (8.55, 13.68)	6.84 (5.13, 10.26)	< 0.001
Albumin (g/L)	43.00 (40.00, 45.00)	43.00 (41.00, 45.00)	41.00 (39.00, 43.00)	< 0.001
Globulin (g/L)	29.00 (26.00, 32.00)	28.00 (26.00, 31.00)	30.00 (28.00, 33.00)	< 0.001
Total protein (g/L)	71.00 (68.00, 74.00)	71.00 (68.00, 75.00)	71.00 (69.00, 74.00)	0.890
Creatinine (µmol/L)	75.14 (63.65, 88.40)	75.14 (63.65, 88.40)	74.26 (62.76, 88.40)	0.132
BUN (mmol/L)	4.64 (3.57, 5.71)	4.28 (3.57, 5.71)	5.00 (3.93, 6.07)	< 0.001
Uric acid (µmol/L)	315.20 (261.70, 374.70)	315.20 (261.70, 374.70)	315.20 (261.70, 380.70)	0.783
Iron (µmol/L)	14.50 (10.90, 18.80)	14.50 (10.90, 18.80)	15.00 (11.50, 19.20)	< 0.001
Sodium (mmol/L)	139.00 (138.00, 141.00)	139.00 (138.00, 141.00)	140.00 (139.00, 142.00)	< 0.001
Potassium (mmol/L)	4.00 (3.80, 4.20)	3.93 (3.73, 4.20)	4.10 (3.80, 4.30)	< 0.001
Chloride (mmol/L)	104.00 (102.00, 105.00)	104.00 (102.00, 106.00)	101.00 (99.00, 103.00)	< 0.001
Bicarbonate (mmol/L)	25.00 (24.00, 27.00)	25.00 (24.00, 26.00)	26.00 (24.00, 27.00)	< 0.001
Calcium (mmol/L)	2.35 (2.30, 2.40)	2.35 (2.30, 2.40)	2.33 (2.28, 2.38)	< 0.001
Phosphorus (mmol/L)	1.20 (1.10, 1.32)	1.20 (1.10, 1.32)	1.16 (1.03, 1.26)	< 0.001
Osmolality (mmol/kg)	278.00 (275.00, 281.00)	278.00 (275.00, 281.00)	281.00 (277.00, 284.00)	< 0.001
WBC count (1000 cells/µL)	6.90 (5.70, 8.40)	6.90 (5.70, 8.30)	7.00 (5.70, 8.40)	0.184
Neutrophils number (1000 cells/µL)	4.00 (3.10, 5.10)	4.00 (3.10, 5.10)	4.00 (3.00, 5.00)	0.046
Neutrophils percent (%)	58.20 (51.80, 64.20)	58.30 (51.90, 64.30)	57.20 (50.80, 63.20)	< 0.001
Lymphocyte number (1000 cells/µL)	2.10 (1.70, 2.50)	2.00 (1.70, 2.50)	2.10 (1.70, 2.60)	< 0.001
Lymphocyte percent (%)	30.30 (25.00, 36.00)	30.20 (24.90, 35.80)	31.20 (25.40, 37.00)	< 0.001
Monocyte number (1000 cells/µL)	0.50 (0.40, 0.70)	0.50 (0.40, 0.70)	0.50 (0.40, 0.70)	< 0.001
Monocyte percent (%)	7.80 (6.40, 9.20)	7.70 (6.40, 9.20)	7.90 (6.70, 9.30)	< 0.001
Platelet count (1000 cells/µL)	241.00 (204.00, 284.00)	241.00 (205.00, 285.00)	236.00 (201.00, 278.00)	< 0.001
Mean platelet volume (fL)	8.10 (7.50, 8.70)	8.10 (7.50, 8.70)	8.20 (7.60, 8.80)	< 0.001
Eosinophils number (1000 cells/µL)	0.20 (0.10, 0.30)	0.20 (0.10, 0.30)	0.20 (0.10, 0.30)	0.184
Eosinophils percent (%)	2.30 (1.50, 3.60)	2.30 (1.50, 3.60)	2.30 (1.50, 3.50)	0.057
Basophils number (1000 cells/µL)	0.00 (0.00, 0.10)	0.00 (0.00, 0.10)	0.10 (0.00, 0.10)	< 0.001
Basophils percent (%)	0.60 (0.40, 0.90)	0.60 (0.40, 0.90)	0.70 (0.60, 1.00)	< 0.001
RBC count (million cells/µL)	4.68 (4.35, 5.02)	4.66 (4.34, 5.01)	4.74 (4.41, 5.07)	< 0.001
Hemoglobin (g/L)	141.00 (131.00, 152.00)	141.00 (131.00, 152.00)	141.00 (132.00, 151.00)	0.027
Hematocrit (%)	41.70 (38.80, 44.70)	41.70 (38.80, 44.70)	42.00 (39.30, 44.60)	< 0.001
MCHC (g/dL)	33.90 (33.30, 34.60)	34.00 (33.30, 34.60)	33.60 (33.00, 34.15)	< 0.001
MCH (pg)	30.50 (29.10, 31.60)	30.50 (29.20, 31.70)	30.00 (28.70, 31.10)	< 0.001
MCV (fL)	89.50 (86.30, 92.60)	89.60 (86.40, 92.70)	88.90 (85.60, 92.05)	< 0.001
RDW (%)	13.00 (12.50, 13.70)	12.90 (12.40, 13.60)	13.50 (13.00, 14.20)	< 0.001

^1^ median (IQR) for continuous; n (%) for categorical.

^2^ Wilcoxon rank‐sum test; chi‐squared test.

**Abbreviations**: BMI: body mass index; HDL: high density lipoprotein; ALP: alkaline phosphatase; ALT: alanine aminotransferase; AST: aspartate aminotransferase; GGT: gamma glutamyl transferase; LDH: lactate dehydrogenase; TB: total bilirubin; BUN: blood urea nitrogen; WBC count: white blood cell count; RBC count: red blood cell count; MCHC: mean corpuscular hemoglobin concentration; MCH: mean corpuscular hemoglobin; MCV: mean corpuscular volume; RDW: red cell distribution width.

There were 7,579 participants (24%) identified as being at potential risk of depression, with the incidence of depression risk in the training and validation cohorts being statistically indistinguishable (24% versus 25%, *p* = 0.177). Significant differences (*p*<0.05) were observed between the training and validation cohorts across a broad spectrum of demographic characteristics, anthropometric measurements, and hematological parameters.

### 3.2 Constructing the Predictive Model in the Training Cohort

**FIGURE 2:**
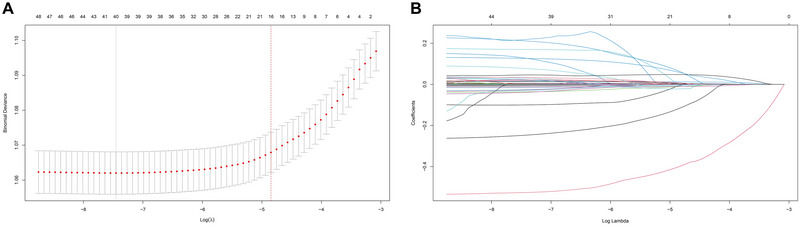
LASSO‐based feature selection for depression risk prediction. (A) LASSO coefficient path plot and (B) cross‐validation curve for optimal parameter selection.

For the construction of the most efficacious predictive model for depression risk, we employed LASSO regression analysis on 50 variables. As illustrated in Figure 2A and Figure 2B, the LASSO screening variable was computed based on the maximum lambda commensurate with the selection error mean within a single standard deviation of the minimum. The lambda value at one standard deviation from the minimum, gleaned from our LASSO regression analysis, was identified as 0.0078. Following covariate screening, sixteen variables (age, gender, race, BMI, triglycerides, HDL, ALP, GGT, TB, albumin, BUN, bicarbonate, WBC count, RBC count, MCV, and RDW) were discerned and consolidated into a risk prediction model (model 1) for depression via multivariate logistic regression. In Model 1, given the *p*‐values of the variables bicarbonate and RBC count exceeding 0.05, they were subsequently removed and then subjected to a multiple logistic regression, leading to the formation of Model 2. Comprehensive parameters of each variable in the predictive model are outlined in Supplementary Table S4.

### 3.3 Evaluating the Predictive Capacity of Three Models for Depression in Training and Validation Cohorts

**FIGURE 3:**
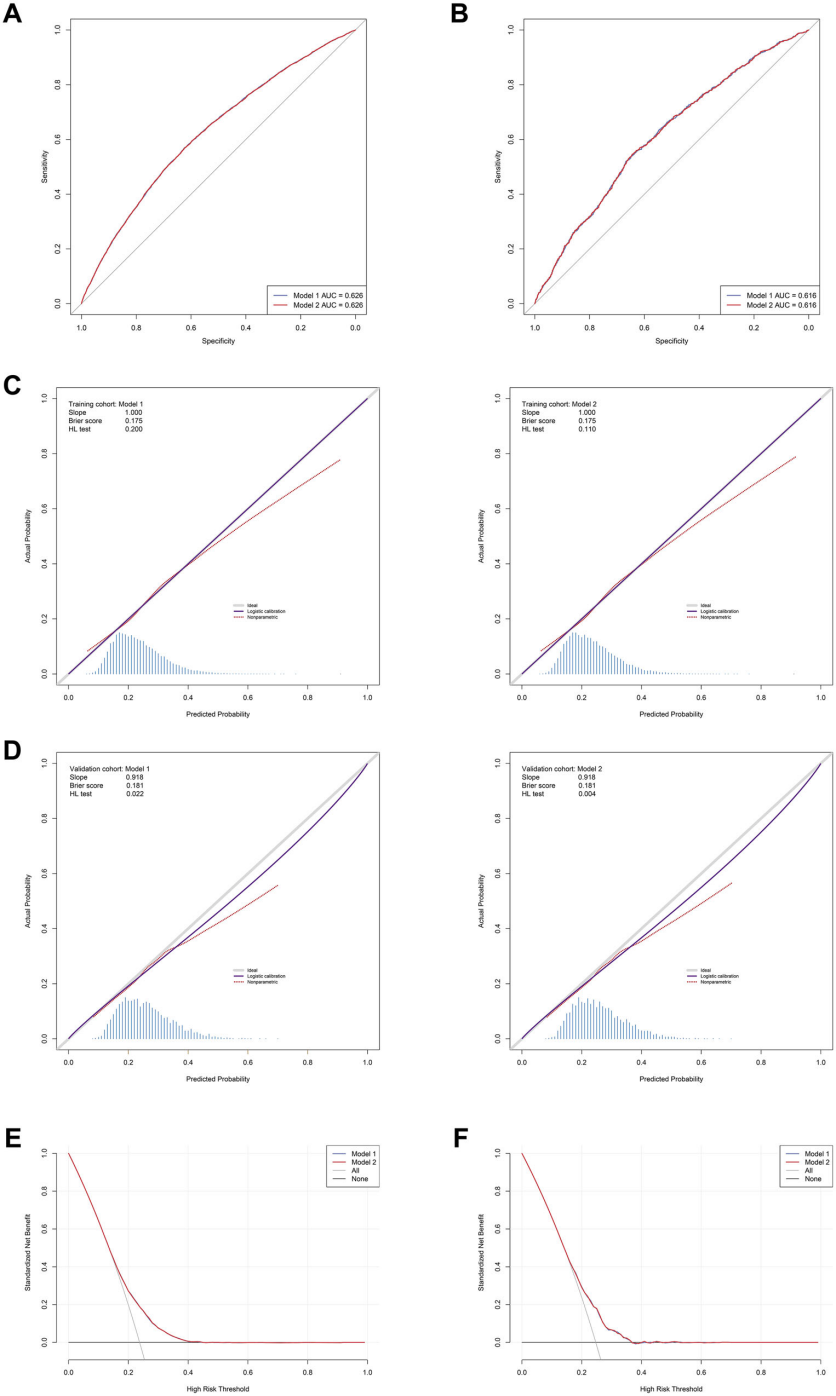
Comparative analysis of model performance metrics between Model 1 and Model 2. (A, B) Receiver operating characteristic curves showing discriminative ability in training and validation cohorts, respectively, (C, D) calibration plots assessing prediction accuracy in training and validation cohorts, respectively, and (E, F) decision curve analysis demonstrating clinical utility in training and validation cohorts, respectively.

ROC analysis of the two models in both training and validation cohorts are displayed in Figure 3A and Figure 3B. Within the training cohort, no statistically significant disparity was noted in the AUC values between model 1 and model 2 (0.626 versus 0.626, DeLong *p*‐value  > 0.05). The AUC values of model 1 and model 2 in the validation cohort were as follows: 0.616 and 0.616, and their corresponding DeLong *p*‐value > 0.05. These findings denote the impressive stability of the two models.

As depicted in Figures 3C and D, the calibration curves delineating the performance of the two examined models across the training and validation cohorts are presented. The models achieved perfect calibration on the training set (slope = 1.0), and maintained strong calibration performance on the validation set with a slope of 0.918, which is remarkably close to the ideal value of 1.0. This consistency between training and validation performance indicates that both models are well‐calibrated and reliable in their probability predictions, showing minimal degradation when generalizing to unseen data. BS values for all two models within both the training and validation cohorts fell beneath the 0.25 threshold, thereby denoting their robust calibration and discriminative abilities. The HL test results showed good calibration in the training cohort (*p* > 0.05). Although the *p*‐values in the validation cohort were 0.022 and 0.004 for model 1 and model 2 respectively, it's important to note that the HL test is known to be sensitive to sample size and can detect even minor deviations in large datasets. Given the visual assessment of calibration curves, slopes close to 1.0, and BS below 0.25, these results suggest that both models maintain clinically acceptable calibration performance.

Supplementary Table S5 delineates the relative performance of the two models, quantified through NRI and IDI. Utilizing the NRI and IDI methodology, the analyses revealed negligible disparities in the classification performance of the two models (all *p* > 0.05).

As exhibited in Figures 3E and F, the DCA implies that utilizing these two models to identify depression could confer a net clinical benefit across both training and validation cohorts when the threshold probability extends approximately from 0.15 to 0.4. The performance of the two models exhibited no discernable difference in the DCA across both the training and validation cohorts.

**FIGURE 4:**
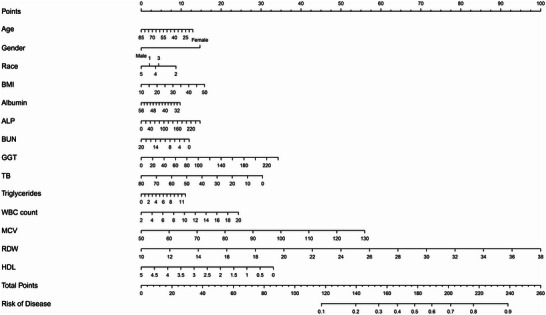
Clinical nomogram for depression risk prediction. Points are assigned for each variable, with total points corresponding to predicted probability of depression. For race categories, 1: Mexican American; 2: other hispanic; 3: non‐hispanic white; 4: non‐hispanic black; and 5: other race.

Through a comprehensive assessment of the preceding statistical methodologies, it can be ascertained that model 2's performance was not markedly inferior to model 1. Furthermore, given Model 2's advantage of a reduced variable count compared to Model 1, it emerged as the superior model. In light of the multivariable logistic regression results deduced from model 2, a nomogram was subsequently developed and is elucidated in Figure 4.

### 3.4 Variable Selection for MR Analysis

In our endeavor to elucidate the causal relationship between an array of variables, including age, gender, race, BMI, triglycerides, HDL, ALP, GGT, TB, albumin, BUN, WBC count, MCV, and RDW, we employed MR analysis (model 2). Recognizing the unavailability of pertinent SNPs associated with gender, testosterone levels were adopted as an apt surrogate, justified by the superior testosterone levels exhibited in men. Due to the unavailability of SNPs associated with age and race, these variables were subsequently excluded from the MR study. Thus, the MR analysis was harnessed to scrutinize the causal interplay between depression and testosterone, BMI, triglycerides, HDL, ALP, GGT, TB, albumin, BUN, WBC count, MCV, and RDW.

### 3.5 Primary MR Findings

**FIGURE 5:**
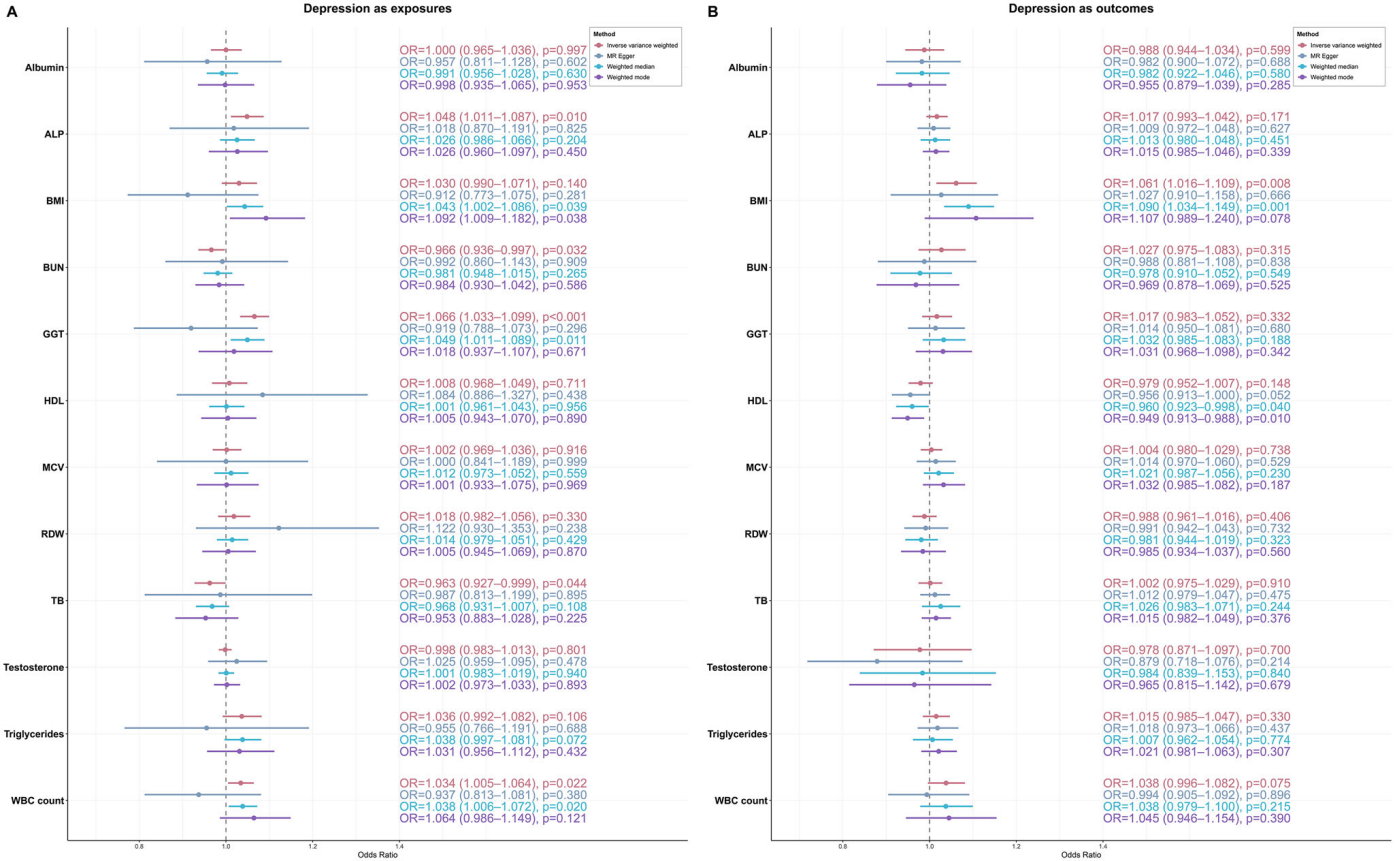
Results of bidirectional two‐sample Mendelian randomization analyses. (A) Causal effects of depression on laboratory parameters and (B) causal effects of laboratory parameters on depression risk.

The results of MR analyses are presented in Figure 5. In analyses where depression was considered as the exposure, IVW analysis demonstrated that depression was causally associated with elevated ALP levels (OR: 1.048, *p* = 0.010) and decreased levels of both BUN (OR: 0.966, *p* = 0.032) and TB (OR: 0.963, *p* = 0.044). Conversely, when depression was analyzed as the outcome, IVW results indicated that higher BMI was causally linked to an increased risk of depression (OR: 1.061, *p* = 0.008).

### 3.6 Sensitivity Analysis Outcomes

The absence of significant pleiotropy is signified by the intercept term of the MR Egger method, which consistently exhibited *p*‐values exceeding 0.05 (Supplementary Table S6). The tests for heterogeneity employed the IVW and MR‐Egger regression, with the *p*‐value of Cochran's Q‐test revealing heterogeneity (*p* < 0.05) (Supplementary Table S7). Despite the presence of heterogeneity, the absence of pleiotropy suggests that the heterogeneity likely stems from other sources rather than pleiotropic effects. Therefore, the IVW findings from the aforementioned MR study retain their credibility.

The sensitivity analyses for MR studies with depression as the exposure and ALP, BUN, and TB as outcomes demonstrated consistent findings. The scatter plots showed directional consistency across different methods, including IVW, MR Egger, weighted median, and weighted mode (Supplementary Figure S1A). The funnel plots revealed no substantial publication bias (Supplementary Figure S1B), while the leave‐one‐out analysis confirmed the stability of results across all SNPs (Supplementary Figure S1C). These comprehensive sensitivity analyses support the robustness of the MR findings for ALP, BUN, and TB as outcome variables.

Similarly, when examining depression as the outcome and BMI as the exposure, the sensitivity analyses yielded equally reassuring results. The scatter plots demonstrated consistent directional effects across all four methods (Supplementary Figure S2A). The funnel plots showed no evidence of significant bias (Supplementary Figure S2B), and the leave‐one‐out analysis confirmed the stability of results across all SNPs (Supplementary Figure S2C). These findings collectively validate the reliability of the MR results with BMI as the exposure variable.

## 4 Discussion

This study introduced a novel depression risk prediction model based on NHANES data and validated through MR analysis. Our model, comprising 14 variables, demonstrated strong predictive capabilities across training and validation cohorts, as evidenced by its calibration, discrimination, and clinical utility metrics. Moreover, MR analyses elucidated bidirectional causal relationships between depression and several biochemical markers, including ALP, BUN, and TB, while affirming the significant influence of BMI on depression risk. These findings underscore the potential for leveraging routine clinical biomarkers in predicting depression, offering a scalable, objective alternative to traditional questionnaire‐based methods.

Our methodological decision to include a comprehensive set of demographic characteristics, anthropometric measurements, and hematological parameters warrants further discussion. This approach was strategically chosen for several reasons. First, the multifactorial nature of depression suggests that single biomarkers alone may be insufficient for accurate risk prediction. Second, the inclusion of multiple variables in our logistic regression model enables more robust variable selection by accounting for potential confounding factors and complex interactions between variables. This comprehensive approach increases the reliability of positive associations identified in our analysis, as variables that maintain statistical significance despite adjustment for numerous potential confounders are more likely to represent genuine biological relationships rather than spurious correlations. Furthermore, while some included parameters may not have direct documented associations with depression, their inclusion serves an important methodological purpose. They act as statistical controls, helping to isolate the truly significant biomarkers from background variation and potential confounding factors. This rigorous approach to variable selection enhances the robustness of our findings and reduces the likelihood of false‐positive associations. The final set of significant biomarkers identified through this comprehensive screening process thus represents a more reliable and potentially more clinically useful set of predictive indicators.

Our investigation yielded two key sets of findings. First, we successfully developed and validated a parsimonious prediction model (Model 2) incorporating 14 readily available clinical variables, which demonstrated robust predictive performance across both training and validation cohorts (AUC 0.626 and 0.616, respectively). The model's stable performance metrics, including consistent calibration curves and favorable decision curve analyses, suggest its potential utility as a screening tool in routine clinical settings. Importantly, the model maintained its predictive capacity despite being more streamlined than the initial version, indicating that the selected variables capture essential aspects of depression risk while remaining practically implementable. Second, our MR analyses revealed several critical bidirectional relationships between depression and biochemical markers. Notably, we identified that elevated BMI levels causally influence depression risk (OR: 1.061, p = 0.008), while depression itself appears to impact several biochemical parameters, including increased ALP levels (OR: 1.048, p = 0.010) and decreased levels of both BUN (OR: 0.966, p = 0.032) and TB (OR: 0.963, p = 0.044). These findings provide strong genetic evidence for the complex interplay between metabolic factors and depression, extending beyond mere association to suggest causal relationships.

Our MR analysis revealed that elevated BMI was causally associated with an increased risk of depression, suggesting that higher body mass index may contribute to the development of depressive symptoms. Numerous epidemiological studies and meta‐analyses have affirmed the correlation between depression and obesity as concurrently occurring medical conditions (McIntyre et al. 2006; Allison et al. 2009; Carey et al. 2014; Faith et al. 2011; de Wit et al. 2010). Numerous factors, such as psychological stress and obesity, can induce inflammation and depressive symptoms (Berk et al. 2013). Obesity is also characterized by systemic inflammation. For instance, central adiposity in obesity serves as a pro‐inflammatory cytokine source, promoting neuroinflammation. Additionally, metabolic perturbations in obesity can augment cortisol, leptin, and insulin levels, precipitating hypothalamic‐pituitary‐adrenal (HPA) axis dysregulation and insulin resistance, which can further potentiate inflammation and exacerbate depression (Hryhorczuk et al. 2013).

Our MR analysis revealed that depression was causally associated with elevated ALP levels, suggesting that individuals with depression may be at risk for increased ALP. Some literature suggests a bidirectional interplay between liver disease and depression, with depression frequently observed as a comorbidity in individuals suffering from liver disease and, conversely, individuals with depression being at an elevated risk for developing liver disease (Ntona et al. 2023; Savage et al. 2023; Cai et al. 2023). ALP, primarily markers of liver function, often signify hepatobiliary disease or damage when elevated. Prior reports have associated increased ALP with psychiatric disorders (Li et al. 2023; Petronijević et al. 2008). Chronic inflammation and heightened oxidative stress may underlie the pathogenesis and progression of both depression and elevated ALP (Labenz et al. 2020; Chan et al. 2019). Depression is associated with several behavioral and physiological changes that may adversely impact liver health, including suboptimal diet, alcohol use, physical inactivity, or dysregulated stress responses. These changes can precipitate metabolic dysregulation, promote systemic inflammation and oxidative stress, and eventually result in liver injury, potentially contributing to the elevation of ALP levels.

Our MR analysis demonstrated that depression was causally associated with decreased TB levels. This finding aligns with previous observational studies that have reported lower TB levels in individuals with depression, including those with comorbid diabetes and postpartum depression (Ye and Wang 2024; Liu et al. 2024). Bilirubin is recognized for its antioxidant and anti‐inflammatory properties, playing a crucial role in neutralizing reactive oxygen species and protecting cells from oxidative damage (Wang et al. 2004; Zhu et al. 2010). Depression has been associated with increased oxidative stress, which could potentially deplete antioxidant reserves, including bilirubin (Miller et al. 2009). This depletion might lead to lower serum bilirubin levels in depressed individuals. Depression is often accompanied by systemic inflammation, characterized by elevated levels of pro‐inflammatory cytokines (Maes et al. 2011). Inflammation can influence liver function and bilirubin metabolism, potentially leading to alterations in bilirubin levels. For instance, inflammatory processes might enhance the conversion of bilirubin to its conjugated form or increase its excretion, resulting in reduced total bilirubin levels (Jayanti et al. 2020). However, the exact mechanisms remain speculative, and further research is needed to clarify this relationship.

Our MR analysis revealed that individuals with depression exhibited significantly lower BUN levels. Urea, the principal constituent of BUN, is a compound synthesized in the liver as a terminal product of protein metabolism (Koo et al. 2004). While the relationship between depression and BUN levels has been previously explored, the underlying mechanisms remain incompletely understood (Feng et al. 2024; Mao et al. 2022). Depression often manifests with altered appetite and dietary patterns, particularly reduced food intake, which may lead to decreased protein consumption. Given that BUN is directly derived from protein metabolism, this reduction in protein intake could explain the observed lower BUN levels in depressed individuals. Supporting this hypothesis, a study by Hu et al. examined 260 hemodialysis patients and found that 10% of participants were diagnosed with depression. Notably, patients with depression demonstrated significantly lower BUN levels compared to those without depression. The authors suggested that depressive symptoms frequently correlate with diminished appetite and poor nutritional status, particularly in hemodialysis patients (Hu et al. 2015).

Our model achieved an AUC of 0.616 in validation cohorts, which, while appearing modest at first glance, warrants careful consideration within the broader context of real‐world applicability and our methodological approach. This performance metric reflects our deliberate choice to prioritize ecological validity by analyzing an inclusive, demographically diverse population sample from NHANES without excluding potentially confounding cases. Many existing studies have reported higher AUC values, but these were often achieved by focusing on narrowly defined or homogeneous populations, excluding complex cases or individuals with comorbidities, or limiting their analysis to depression risk prediction in single‐disease cohorts (Zheng et al. 2024; Lin et al. 2021; Samsel et al. 2024). Additionally, several depression prediction models rely on electronic health records or wearable devices for monitoring, which introduce practical challenges and implementation barriers in real‐world settings (Nickson et al. 2023; Sun et al. 2023; Adler et al. 2024). While such selective sampling approaches and the use of advanced monitoring devices may yield more impressive statistical metrics, they potentially compromise the generalizability of findings to clinical settings where patient populations are inherently heterogeneous and complex. Our approach, in contrast, emphasizes practical utility and broad applicability over artificially inflated performance metrics. Furthermore, a key advantage of our model lies in its reliance on accessible and practical indicators, distinguishing it from previous approaches. While existing models often depend on specialized biomarkers, neuroimaging data, or complex psychological assessments that require specific equipment, expertise, and dedicated clinical visits (Kambeitz et al. 2017; Kabbara et al. 2022; Zhang et al. 2024), our model strategically utilizes routine markers commonly obtained during standard health check‐ups. This deliberate selection of variables ensures seamless integration of our screening tool into existing healthcare workflows without the need for additional specialized testing, equipment, or clinical resources. We contend that the practical benefits of utilizing these readily available indicators outweigh the potential marginal improvements in AUC that might be achieved through more specialized, yet less accessible measurements. In the context of initial screening tools for complex conditions like depression, an AUC of 0.616 represents a meaningful improvement over chance and can provide valuable clinical utility when used as part of a comprehensive assessment strategy. Our model is designed to serve as a scalable, cost‐effective screening mechanism in primary care settings, where the ability to implement widespread screening often takes precedence over achieving perfect predictive accuracy. Rather than viewing this as a standalone diagnostic tool, our model should be considered as one component in the broader toolkit for depression screening, offering an objective complement to existing psychological assessment methods.

It is important to acknowledge several limitations of our study. First, our reliance on PHQ‐9 scores rather than formal clinical diagnoses of major depressive disorder (MDD) represents a notable limitation. While PHQ‐9 is a well‐validated screening tool with high sensitivity and specificity, it measures depressive symptoms over only a two‐week period and cannot replace comprehensive clinical diagnosis, including detailed patient history, interviews, and professional psychiatric evaluation. However, this aligns with our primary goal of developing a screening tool for initial risk identification rather than definitive diagnosis. The inherent characteristics of the NHANES database present additional limitations. Although NHANES provides a large, representative sample with temporally consistent psychological and physiological measurements, it does not differentiate between depression subtypes. Depression is a heterogeneous disorder with distinct clinical presentations, biological mechanisms, and treatment responses. Our model's treatment of depression as a unified entity, while practical for broad population screening, may obscure important biological distinctions identified in recent research, such as the distinct subgroups of depressed patients with different patterns of HPA axis activity and inflammatory markers demonstrated in the NESDA study (Beijers et al. 2019). Regarding our MR analysis, while we employed multiple robust methodological approaches, including MR‐Egger regression, weighted median methods, and comprehensive sensitivity analyses, the complete elimination of horizontal pleiotropy and heterogeneity cannot be absolutely guaranteed. The interpretation of our MR findings requires careful consideration of the complex biological pathways, particularly the shared physiological systems that may influence both depression and peripheral markers. For example, the mineralocorticoid receptor system's dual functionality in both peripheral tissues and the central nervous system creates parallel pathways that could affect both depression and our measured biomarkers (Murck et al. 2020, Murck et al. 2019). Finally, although we utilized standardized clinical markers to enhance model replicability, external validation studies are essential to confirm the stability and reliability of our findings across different clinical contexts and demographic groups. Future research should focus on prospective validation studies in diverse clinical settings, potentially incorporating longitudinal data and more refined pleiotropy assessments to evaluate predictive stability over time and elucidate complex biological relationships.

## 5 Conclusion

This research advances depression prediction by utilizing NHANES data and MR analysis. Our predictive model demonstrates potential for large‐scale population screening, offering both improved understanding of depression etiology and early risk assessment capabilities. Such early detection could facilitate timely interventions, potentially reducing the societal and personal burden of depression.

## Author Contributions

**Lin Lin**: methodology, data curation, investigation, formal analysis, visualization, writing–original draft. **Liqun Zhang**: data curation, investigation, visualization. **Jingdong Zhang**: validation. **Dapeng Ding**: conceptualization, validation, project administration, supervision.

## Ethics Statement

This study is a secondary analysis of data from the NHANES, which is publicly available through the Centers for Disease Control and Prevention (CDC). The NHANES program operates under approval from the National Center for Health Statistics Research Ethics Review Board. As per NHANES guidelines, all participants provided informed consent during the original data collection process. Our research exclusively utilized de‐identified, publicly accessible data from the NHANES database. According to the policy for secondary use of public data, this study does not require additional ethical approval or informed consent from participants. The use of this data for research purposes is in compliance with the NHANES data use agreement and relevant data protection regulations.

## Peer Review

The peer review history for this article is available at https://publons.com/publon/10.1002/brb3.70674

## Supporting information




**Supplementary Figures**: brb370674‐sup‐0001‐Figure.docx

**Supplementary Tables**: brb370674‐sup‐0002‐Tables.xlsx

## Data Availability

The datasets analyzed during the current study are publicly available in the NHANES repository (https://www.cdc.gov/nchs/nhanes/index.htm). All relevant processed data and supplementary materials used in this study are available in the supplementary files. Additional data used and analyzed during the current study are available from the corresponding author upon reasonable request.
