# Supplementary material for: A Novel Depression Risk Prediction Model Using NHANES Data With Mendelian Randomization Validation
Source: Brain Behav. 2025 Jul 25;15(7):e70674. doi: 10.1002/brb3.70674 (PMC12409830; doi:10.1002/brb3.70674)
Supplement: Supplementary file 1 — Supplementary Figures: brb370674‐sup‐0001‐Figure.docx [file BRB3-15-e70674-s002.docx]

Figure S1. Mendelian randomization sensitivity analyses for depression as exposure. (A) Scatter plots of genetic associations. (B) Funnel plots for assessment of directional pleiotropy. (C) Leave-one-out plots evaluating robustness of causal estimates.





Figure S2. Mendelian randomization sensitivity analyses for depression as outcome. (A) Scatter plots of genetic associations. (B) Funnel plots for assessment of directional pleiotropy. (C) Leave-one-out plots evaluating robustness of causal estimates.
